# The Management of Scar Ectopic: A Single-Center Experience

**DOI:** 10.7759/cureus.15881

**Published:** 2021-06-23

**Authors:** Neha Agarwal, Shalini Gainder, Seema Chopra, Minakshi Rohilla, GRV Prasad, Vanita Jain

**Affiliations:** 1 Obstetrics and Gynecology, Post Graduate Institute of Medical Education and Research, Chandigarh, IND; 2 Obstetrics and Gynecology, Maulana Azad Medical College, New Delhi, IND

**Keywords:** cesarean scar ectopic pregnancy, methotrexate, uterine artery embolization, b-hcg, csp, kcl, doppler, laparotomy, hysterectomy, ultrasound

## Abstract

Purpose: This study aimed to highlight the clinical features, diagnosis, and different modalities of the treatment of cesarean scar pregnancy (CSP).

Methods: This study was done in the tertiary referral hospital of India for one year. A total of 11 cases were enrolled prospectively. In each case, the diagnostic ultrasonography and measurement of baseline beta-human chorionic gonadotropin (β-HCG) levels were done. The treatment was given based on the hemodynamic status of the patient and desire for future fertility. Various treatment modalities used were medical, surgical, or interventional digital subtraction angiography to control hemorrhage. Also, in some cases, ultrasound-guided methotrexate was injected into the scar ectopic. Medically treated cases were followed up until their β-HCG levels became normal.

Results: Out of 11 patients, six had a history of two cesarean sections in the past, four patients had a history of one cesarean section and one patient with a previous three low segments cesarean section (LSCS). Seven out of 11 patients underwent medical management with either methotrexate with potassium chloride (KCl) or methotrexate alone. The success of the medical management was monitored by serial β- HCG values. The mean time for the resolution of these 10 patients was 86.7 ± 53.6 days. Three patients underwent emergency uterine artery embolization due to uncontrolled bleeding and one patient required laparotomy.

Conclusion: CSP is a life-threatening condition that can be diagnosed with the help of transvaginal ultrasonography. The treatment, however, depends on the hemodynamic status of the patient and desire for future fertility. Well-defined diagnostic criteria coupled with structured management and follow-up protocol can help in treating this challenging form of ectopic pregnancy.

## Introduction

Implantation of the pregnancy within the scar of the previous cesarean section can be a life-threatening condition. Cesarean scar pregnancy (CSP) was first described in 1978 by Larsen and Solomon [1]. Awareness about the complications and modes of management of CSP needs to be created. With the increasing incidence of cesarean deliveries, obstetricians need to be well versed with this rare phenomenon. In CSP, the gestation sac is surrounded by myometrium and the fibrous tissue of the scar [2]. The mechanism of the scar implantation is attributed to the invasion of the myometrium through a microscopic tract between the cesarean section (CS) scar and the endometrial canal [3,4]. The incidence of CSP has been estimated to range from 1/1800 to 1/2500 of all cesarean deliveries performed [5].

The patients with CSP can have variable clinical profiles. They may present as spontaneous abortion in the first trimester or with devastating complications like uterine rupture, adherent placenta, and hemorrhagic shock in the second or third trimester. The main objective of our study is to highlight the clinical features, diagnosis, and different modalities of the treatment of CSP with a background of our clinical experience. We describe the management of patients with CSP based on their varied clinical presentations.

## Materials and methods

This study was carried out in the department of Obstetrics & Gynecology of Post Graduate Institute of Medical Education and Research (PGIMER), Chandigarh, India, for a total of one year. A total of 11 cases were enrolled, six prospectively and five retrospectively. The patients were diagnosed with CSP based on the grayscale transvaginal ultrasound scan. We analyzed the data from the inpatient records related to presenting complaints, maternal age, gravidity, gestational age (weeks), number of previous cesarean section, previous history of abortion, presenting complaints, presence of fetal cardiac activity in an ultrasound scan, history of medical termination of pregnancy (MTP) intake and history of dilatation and curettage in the current pregnancy. The diagnostic ultrasound and the measurement of baseline human chorionic gonadotropin (β-HCG) levels were done in each case. 

The treatment was given based on the hemodynamic status of the patient, gestational age, and desire for future fertility. The various treatment paradigms used were medical, surgical, and uterine artery embolization (UAE) via angiography. The medical options offered were dependent on the presence of fetal cardiac activity. In presence of fetal cardiac activity, injection potassium chloride (1-2 mEq) was instilled into the fetal heart using a 20 G, 15 cm long needle under transvaginal ultrasound guidance. This was followed by the administration of systemic methotrexate (dose calculated as per body surface area). Serial monitoring of β-HCG levels was done, on days one, seven, 14, 21, and further to document the subsequent fall in serum levels.

In the absence of cardiac activity, the patient received a methotrexate injection. Subsequent monitoring of β-HCG levels was carried out till serum levels normalized. If the patient presented with hemodynamic instability, resuscitation was performed till adequate circulatory volume status was restored. A further decision was taken based on the patients’ wish to preserve fertility. A laparotomy along with a hysterectomy was performed if the patient did not wish to retain fertility.

In the case of an actively hemorrhaging patient, angiographic assisted treatment is life- and time-saving. The embolization of the bilateral uterine arteries aided by digital subtraction angiography was done by the intervention radiologist. Clearance from the ethics committee of the hospital was taken.

## Results

A total of 11 patients were included in the study. Table 1 below shows the clinical characteristics of the patients included in the study. The mean age of the study population was 31.5 ± 5.2 years. The mean gestation age was 11.4(± 4.18) weeks. All these patients had conceived spontaneously. A total of 81.8% of the women complained of bleeding per vaginum (n=9/11). In all the cases, transvaginal ultrasonography along with serum β-HCG analysis was used as diagnostic tests for CSP.

**Table 1:** The Clinical Details of 11 Patients NPOL - non-progression of labor, LSCS - low segment cesarean section, ICP - interconceptional period, BOH - bad obstetric history, PV - per vaginum

Case No.	Maternal Age (yrs.)	Gravida (G) and Parity (P)	Gestation Age (weeks)	Number of Previous LSCS	Indication of Previous LSCS	Previous History of Abortion	Presentation	Fetal Cardiac Activity	MTP Pill	D and C
1	25	G3P1011	7	1	Term NPOL	0	Asymptomatic	Present	No	No
2	35	G4P2021	12	2	Term - Fetal distress Term - Previous LSCS	2	Bleeding PV	Absent	Yes	Yes
3	27	G2P1001	6	1	Term - breech	0	Bleeding PV	Present	No	No
4	33	G4P2011	11	2	Term – Abruption @ 36 weeks - Short ICP	1	Bleeding PV	Present	No	No
5	35	G5P2022	17	2	Term - Breech Term - Previous LSCS	2	Bleeding PV	Absent	Yes	No
6	35	G5P4001	18	1	Term - BOH	0	Bleeding PV	Absent	No	Yes
7	34	G4P3001	11	3	Term - Placenta Praevia Term - Previous LSCS Term - Previous 2 LSCS	0	Bleeding PV	Absent	Yes	No
8	26	G2P1001	8	1	Term - Fetal Bradycardia	0	Pain at the scar site	Present	No	No
9	23	G3P2002	15	2	Term - NPOL Term - Previous LSCS Bladder Repair	0	Bleeding PV	Absent	Yes	No
10	39	G3P2002	14	2	Term - Fetal Bradycardia Term - Previous LSCS	0	Bleeding PV	Absent	No	Yes
11	35	G3P2002	7	2	Term - Failed Induction Term - Previous LSCS	0	Bleeding PV	Absent	Yes	No

Table 2 depicts the primary treatment given to the included patients and their subsequent outcomes. Twenty-seven percent of the patients (cases 1, 3, and 4) had evidence of fetal cardiac activity. These were treated by the intracardiac instillation of 1-2 mEq potassium chloride (KCl) followed by systemic methotrexate therapy. Sixty-six percent of these developed excessive vaginal bleeds on day 77 (case 1) and day 45 (case 3) after treatment. They were further diagnosed to have a uterine arteriovenous malformation (AVM) based on angiographic studies and subsequently underwent uterine artery embolization. Thirty-six percent of the patients (cases 2, 7, 8, and 11) were administered only systemic methotrexate, as there was no evidence of fetal cardiac activity. Seventy-five percent of patients (n=3/4) had a history of prior consumption of MTP pills. Twenty-five percent of the patients (case 2) presented with hemorrhage on day 43 after the treatment and underwent embolization in view of profuse vaginal bleeding. Twenty-seven percent of patients (three out of 11) initially presented with incomplete abortion on intake of MTP pills at the nearby clinic and dilatation and curettage was attempted. Subsequently, they were referred to our emergency services in view of acute hemorrhage. Further ultrasonographic imaging had confirmed the diagnosis of scar pregnancy. After initial resuscitation, they underwent emergency uterine artery embolization. Only one patient (case 5) underwent emergency laparotomy followed by hysterectomy because of massive bleeding in the late second trimester where the placenta was adherent, and the fetus was occupying the lower uterine cavity with the empty fundus of the uterus. The treatment and outcome of these patients are listed in Table 2.

**Table 2:** Treatment and Outcome of the Patients MTX - methotrexate, KCl - potassium chloride, AVM - arteriovenous malformation

Case No.	Primary Treatment	Success	Secondary Treatment/Maternal Outcome
1	Intracardiac KCl + systemic MTX (2 doses)	Yes	Uterine AVM Embolization
2	Systemic MTX (2 doses)	Yes	Uterine AVM Embolization
3	Intracardiac KCl + systemic MTX (2 doses)	Yes	Uterine AVM Embolization
4	Intracardiac KCl + systemic MTX (2 doses)	Yes	None
5	Laparotomy followed by Hysterectomy	Yes	None
6	Uterine Artery Embolization	Yes	Gluteal Skin Necrosis
7	Systemic MTX (2 doses)	Yes	None
8	Systemic MTX (3 doses)	Yes	None
9	Uterine Artery Embolization	Yes	None
10	Uterine Artery Embolization	Yes	None
11	Systemic Methotrexate	Yes	None

The patients receiving medical management were followed based on serum β-HCG levels measured on day four and day seven and then every week until the serum levels were undetectable. The patients undergoing UAE had a follow-up with weekly β-HCG levels. β -HCG value for each patient is listed in Table 3. In addition, an ultrasound was also done to look for the reduction in the size of the gestation sac.

**Table 3:** Pre- and Post-Treatment β-HCG Value and Days of Resolution NA - not applicable, β-HCG - beta-human chorionic gonadotropin

Case No.	Pre-treatment β-HCG values (mIU/ml)	Post-treatment β-HCG values (mIU/ml)	Days of Resolution
1	56,860	0.1	106
2	63,560	<3	95
3	7614	3.02	61
4	38,444	<3	92
5	NA	NA	NA
6	12093.2	<1.2	24
7	5135	<0.1	218
8	29081.1	0.54	63
9	900	0.4	43
10	2257	0.67	87
11	9000	1100	Lost to follow-up

Overall in our study, seven patients (63.6%) underwent medical management with methotrexate with or without intracardiac injection of potassium chloride. Three patients (27%) underwent emergency uterine artery embolization and one (9%) underwent a hysterectomy.

## Discussion

CSP is a life-threatening condition and carries a high risk of uterine rupture and massive hemorrhage. Despite the medical description of CSP as early as 1978, its diagnosis and management remain unclear. This is due to the rarity of the scar pregnancy. Our case series represents one of the largest case series from India. In our study, we found that the symptoms of cesarean scar ectopic pregnancy are like that of abortion. This frequently leads to misdiagnosing these patients as incomplete abortions and ultimately undergo blind procedures like dilatation and curettage.

The reported mean age group in one of the largest case series reported from Israel was 35 years, whereas, in our study, it was 31.5 years [6]. It may present from as early as five to six weeks to as late as 16 weeks [7,8]. Although, in our study, there was one patient who was diagnosed with scar ectopic at as late as 18 weeks of gestation. The most frequent presenting complaint is the painless vaginal bleed (39%) and so was in our group of patients (81.8%) [9]. With the rising rate of cesarean deliveries worldwide, the incidence of CSP is increasing as well [7,10]. Maymon et al. found that 50% of the CSP patients underwent multiple cesarean sections while Jurkovic et al. have found that 72% of their patients underwent multiple (≥2) cesarean sections [6,11]. In our study, the rate of multiple cesarean sections was 63.6% which is like the previous studies. Prior multiple cesarean deliveries predispose patients to develop scar pregnancy. This may be due to the shortening of the viable scar-free uterine segment available for implantation. The conceptus develops in the proximity of the previous scar and usually encompasses the region of scarred myometrium within the developing wall.

Apart from the age and previous cesarean section, the relationship between gravidity, parity, and previous abortion with CSP has been well-documented. A study conducted by Zhou et al. reported that gravidity and previous induced abortions were independent risk factors for developing CSP [12]. Our study also showed an increased risk of CSP with an increase in gravidity (≥ 3) as 81% of our patients fell in this category.

**Figure 1:**
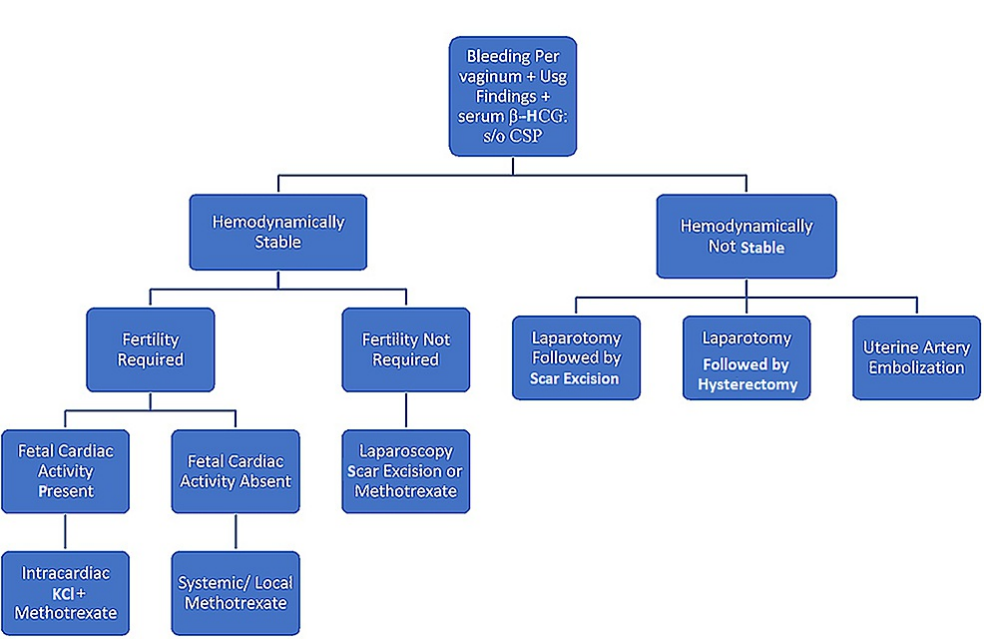
Proposed Algorithm for the Management of Scar Ectopic KCl - potassium chloride, β-HCG - beta-human chorionic gonadotropin

Ultrasound is considered the first line of diagnostic modality to diagnose CSP. There are four diagnostic features seen in the transvaginal ultrasound vis-a-vis (i) an empty uterus, (ii) an empty cervical canal, (iii) discontinuity in the anterior uterine wall, and (iv) the gestational sac located in the anterior part of the isthmic portion of the uterus with a diminished myometrial layer between the bladder and the sac [3-5]. The sensitivity of transvaginal ultrasonography in diagnosing CSP is 86.4% [7]. The diagnosis is based on the presence of a gestational sac at the site of the previous cesarean scar in the presence of an empty uterine cavity and cervix. A thin myometrial layer may be seen adjacent to the bladder wall in the antenatal ultrasound [5]. A proposed algorithm for CSP diagnosis and management is given in Figure 1.

The treatment of CSP depends on the clinical presentation of the patient. If the patient has massive bleeding or uterine rupture, then emergency embolization or laparotomy followed by scar excision or hysterectomy is indicated. The desire to retain fertility is of paramount importance here. In our study, four patients (36.3%) presented with life-threatening bleeding, where three underwent emergency bilateral uterine artery embolization and one needed an emergency hysterectomy. It is particularly important to mention that three out of these four patients who presented with acute bleeding were initially managed with dilatation and curettage (D&C). There could be a possibility that D&C not just failed to manage the initial disease but may have led to the development of arteriovenous malformation. The laparoscopic excision of the previous scar is a surgical option that needs to be considered in women without living issues. The main advantage of laparotomy or laparoscopy is quick normalization of β-HCG values and decreasing the risk of recurrence as it involves complete removal of the microtubular tract [13].

The medical treatment is primarily indicated in hemodynamically stable patients. The aim of medical management is avoidance of laparotomy and preservation of fertility. However, close surveillance with β-HCG level is essential to monitor the response. The medical management includes the use of methotrexate either local or systemic with or without the use of potassium chloride. The rationale for injecting potassium chloride after the confirmation of the diagnosis is to immediately cause fetal death and prevent further invasion and proliferation of villi. Seow et al. have previously advocated the use of ultrasound-guided methotrexate in the successful management of scar ectopic [8]. A lower β-HCG value and greater than 2 mm myometrial thickness between the gestation sac and the bladder appears to increase the success of methotrexate [14]. Although, we did not determine any threshold of β-HCG while formulating the treatment. The success of the medical management is monitored by serial β-HCG values and is recommended until the value reaches < 5 mIU/ml [7].

The mean time for the resolution of β-HCG in these 10 patients was 86.7 ± 53.6 days. In the study by Timor-Tritsch et al., the mean resolution of β-HCG values excluding patients undergoing hysterectomy or embolization was 88.6 days [5].

In the past, many investigators have advocated methotrexate as the first-line management of scar pregnancy. Previous studies have shown that 50% of patients with CSP initially managed with methotrexate would require additional intervention [11,15]. In our series, seven patients received medical therapy. Three among them (42.8%) developed uterine AVM as a complication. Timor-Tritsch et al. had reported complications in 54 patients out of 87 (62.1 %) receiving methotrexate as the primary modality of treatment [5]. The complications in this study included both immediate as well as delayed, which required a secondary intervention. Ilan et al., in the past, have emphasized the relationship between uterine arteriovenous malformation and CSP [16]. According to them, methotrexate treatment would result in slow resolution of scar pregnancy. Hence, technically there is a retained product of conception in the uterus which can further increase the possibility of developing uterine AVM in these patients.

When uterine artery embolization was used as the primary modality of treatment in our study, no complications related to CSP were reported. Only one patient had gluteal skin necrosis, which was managed symptomatically. The viability of the post-embolised uterus is something that needs to be studied in detail since the option of fertility preservation also needs to be considered in patients who may be surgical candidates.

In our series, life-threatening vaginal bleeding was documented in all patients who underwent dilatation and curettage. So, we believe that curettage should not be used as a primary modality of treatment in CSP. We observed that systemic methotrexate is an effective primary modality in the treatment of CSP with fewer complications. However, this seems to be acceptable only in the absence of fetal cardiac activity. The possible explanation could be slow absorption or activity of the drug which may fail to inhibit the vascularisation of the growing fetus resulting in secondary bleeding. In the presence of fetal cardiac activity, the beneficial role of intra-amniotic injection of methotrexate along with potassium chloride is well documented in the literature [11]. Timmerman has suggested the use of peak systolic velocity of the uterine vessels in stratifying the patients who are likely to develop arteriovenous malformation following the scar pregnancy [17]. 

Also, we did not leave any patient for the expectant management, however, there have been reports in the past that this could be a viable option for the scar ectopic with no fetal cardiac activity [18]. Given the high risk of uterine rupture and vigilant monitoring, we do not suggest it to be the first line of management.

The higher β-HCG levels are found in most cases of cesarean scar pregnancy and this should not be considered as an inhibition to resort to the non-surgical methods in women desiring future fertility. The women should be counseled about the likely success of the procedure, the possibility of acute hemorrhage during the follow-up, and the slow resolution of the visible scar pregnancy on the ultrasound.

The limitation of our study is that we did not use the doppler indices for predicting the outcome of the patient. The number of the recruited patients was less given the rarity of this condition. 

## Conclusions

CSP is a rare yet life-threatening obstetric condition. It can be diagnosed early with the help of transvaginal ultrasonography complemented with doppler. The treatment is not well defined, yet we suggest the use of medical management as the first line of treatment in hemodynamically stable patients. Invasive procedures like laparotomy or embolization should be reserved for the acutely bleeding patient. β-HCG should not be the determining factor in the management of this rare form of ectopic pregnancy. Well-defined diagnostic criteria coupled with structured management and follow-up protocol can help in treating this challenging form of ectopic pregnancy.
